# Integrated multi-omics analyses reveal that *BCAM* is associated with epigenetic modification and tumor microenvironment subtypes of clear cell renal cell carcinoma

**DOI:** 10.1186/s13148-022-01319-2

**Published:** 2022-08-08

**Authors:** Junjie Zhao, Jiayu Liang, Yang Yang, Guangxi Sun, Xingming Zhang, Jinge Zhao, Xu Hu, Junru Chen, Sha Zhu, Yuchao Ni, Yaowen Zhang, Jindong Dai, Zhipeng Wang, Zilin Wang, Yuhao Zeng, Jin Yao, Ni Chen, Pengfei Shen, Zhenhua Liu, Hao Zeng

**Affiliations:** 1 Department of Urology, Institute of Urology, West China Hospital, Sichuan University, No.37 Guoxue Alley, Wuhou District, Chengdu City, 610041 Sichuan Province People’s Republic of China; 2 Department of Radiology, West China Hospital, Sichuan University, Chengdu, 610041 People’s Republic of China; 3 Department of Pathology, West China Hospital, Sichuan University, Chengdu, 610041 People’s Republic of China

**Keywords:** BCAM, Clear cell renal cell carcinoma, Methylation, Immune microenvironment, Angiogenesis

## Abstract

**Background:**

Clear cell renal cell carcinoma (ccRCC) is the most common and highly heterogeneous subtype of renal cell carcinoma. Dysregulated basal cell adhesion molecule (*BCAM*) gene is associated with poor prognosis in various cancers. However, the dysregulated functions and related multi-omics features of *BCAM* in ccRCC stay unclear.

**Results:**

*BCAM* expression was aberrantly downregulated in ccRCC and correlated with adverse pathological parameters and poor prognosis. Low mRNA expression of *BCAM* was remarkably associated with its CpG methylation levels and BAP1 mutation status. Patients with lower-expressed *BCAM* concomitant with BAP1 mutation had a worse prognosis. Using RNA-seq data from The cancer genome atlas, we found that compared to the *BCAM*-high expression subgroup, ccRCC patients in the *BCAM*-low expression subgroup had significantly higher levels of immune infiltration, higher immune checkpoint expression levels and lower TIDE (tumor immune dysfunction and exclusion) score, indicating potential better response to immunotherapy. Data from the Clinical Proteomic Tumor Analysis Consortium further validated the association between low *BCAM* expression and CD8 + inflamed phenotype at protein level. Meanwhile, our results suggested that the angiogenesis-related pathways were enriched in the *BCAM*-high expression subgroup. More importantly, according to the data from the GDSC database, we revealed that the *BCAM*-high expression subgroup should be more sensitive to anti-angiogenetic therapies, including sorafenib, pazopanib and axitinib.

**Conclusions:**

These results suggest that *BCAM* could serve as a biomarker distinguishing different tumor microenvironment phenotypes, predicting prognosis and helping therapeutic decision-making for patients with ccRCC.

**Supplementary Information:**

The online version contains supplementary material available at 10.1186/s13148-022-01319-2.

## Background

Renal cell carcinoma (RCC) is the most common form of kidney cancer, accounting for up to 85% of the cases [1]. According to its diverse morphologies and specific driver gene alterations, RCC has been subclassified into at least 12 subtypes [2]. Among them, clear cell RCC (ccRCC), papillary RCC (pRCC) and chromophobe RCC (chRCC) are the three major subtypes presenting more than 90% of RCC [3]. Despite the improvement in health screening and imaging techniques, more and more RCC can be diagnosed early and thus become curable with radical surgery. However, till now, 30–40% of RCC unavoidably develop into metastatic diseases, requiring systemic therapies [4]. In the past three decades, the median overall survival of metastatic RCC has improved from less than 1 year to more than 4 years with novel immunotherapy and targeted therapy, with a deep understanding of molecular events [5, 6]. However, the conflict between tumor heterogeneity and personalized therapeutic strategy remains unmet and, to some extent, limits the efficacy of these novel agents. Novel predictive nomograms or biomarkers are expected to predict prognosis and optimize therapeutic decision-making. There have been relevant reports on molecular types of RCC, for example, PD-L1 and PBRM1 [7, 8]. However, in general, whether these genes could serve as precise biomarkers to classify therapeutic strategies for ccRCC patients remains controversial [9, 10]. Therefore, it is necessary to explore new and competent biomarkers.

Basal cell adhesion molecule (*BCAM*) was a 90 kDa membrane-bound glycoprotein of the immunoglobulin superfamily (IgSF), functioning as a receptor for the extracellular matrix protein, laminin [11]. Growing evidence has demonstrated the association of *BCAM* with different cancers. BCAM is differentially expressed in some tumors: highly expressed in tumors like epithelial skin, ovarian, bladder and gastric cancer [12–15] and downregulated in some other types of malignancies (Table 1) [16, 17]. Mechanistically, aberrant expression of cell adhesion molecules facilitates tumor metastasis by disrupting normal cell–cell and cell–matrix interactions, including the IgSF members [18]. *BCAM*, involved in cell adhesion and migration, can also promote tumor metastasis and has been elucidated to play a functional role in the metastasis of thyroid cancer and gastric cancer [15, 16]. However, the expression profile and underlying mechanisms of *BCAM* in RCC tumorigenesis remain unknown. What is noteworthy is that several IgSF members can also mediate the formation of tumor aggregates and protect the inner cells from the cytotoxic activity of the immune system, eventually leading to tumor immune evasion [18]. Considering the diverse immune infiltration patterns and various expressions of immune evasion biomarkers, such as immune checkpoints, in RCC, it is important to explore the association between *BCAM* and tumor immune cell infiltration in RCC.

**Table 1:** Correlation between BCAM expression and clinicopathological features in different cancers

Types	BCAM expression level	Stage	Grade	Prognosis
Epithelial skin tumor	Upregulated	–	–	–
Ovarian cancer	Upregulated	–	–	–
Bladder cancer	Upregulated	Positive	No significant	No significant
Gastric cancer	Upregulated	–	–	Positive
Thyroid cancer	Downregulated	–	–	–
Colon cancer	Downregulated	–	–	–

Few studies focus on the regulatory mechanism of abnormal expression of *BCAM* in tumors, except one reported that *BCAM* expression was modulated by lncRNA BAN in gastric cancer [15]. Epigenetics comprises specific heritable DNA and chromatin signatures that have an important bearing on the establishment and maintenance of correct transcription procedures in specific cell lineages, and of its hallmarks, posttranslational modifications of histones and DNA/RNA methylation are of the most significance. Mutations of known tumor suppressor genes, for example, VHL, PBRM1 and BAP1, are commonly observed in ccRCC. In addition to the somatic mutations observed in RCC, during tumorigenesis, the number of genes found to be inactivated through epigenetic modifications increases continuously, encompassing DNA/RNA methylation and abnormal histone modifications [19]. Hence, the epigenetic modification was also crucial in the abnormal regulation of genes resulting in RCC, besides gene mutation. However, to our knowledge, there is no report about the potential epigenetic modification of *BCAM* in RCC.

In this study, based on multi-omics data, we first discussed the differential expression of *BCAM* in RCC and its relationship with the prognosis, observed that dysregulation of *BCAM* could be associated with genetic and epigenetic modification, and further found that low expression of *BCAM* was related to the enrichment of immune infiltration, implying the potential favorable response to immunotherapy among ccRCC. The results not only provide a new basis for understanding the role of *BCAM* in developing RCC, but also found that the expression of *BCAM* could help classify different multi-omics molecular types and even make optimal therapeutic strategies decision.

## Results

### The expression of BCAM was abnormally downregulated in RCC

**Fig. 1:**
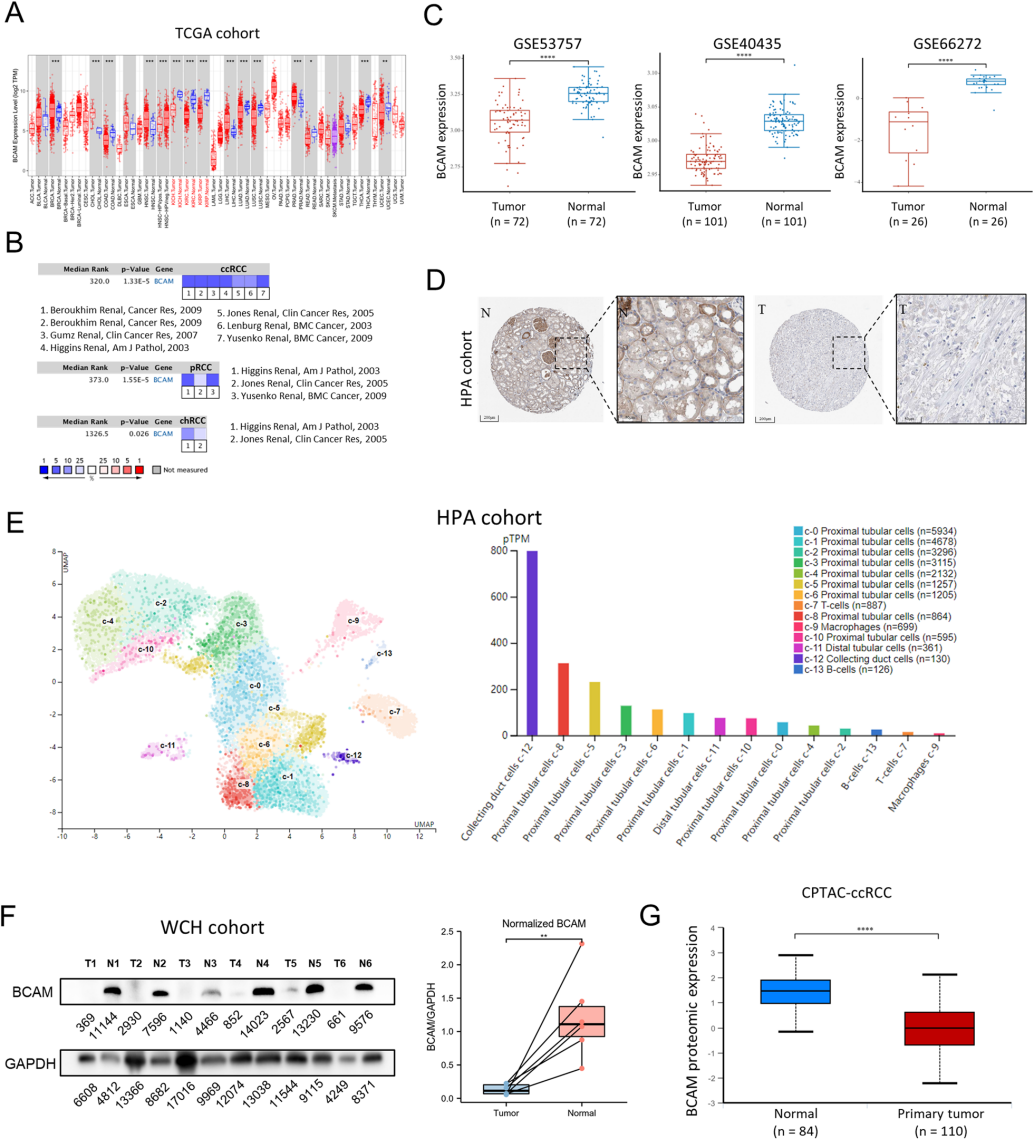
*BCAM* mRNA and protein expression was lower in RCC tissues than in normal kidney tissues. **A** *BCAM* mRNA expression in tumor and normal tissues from pan-cancer data of The Cancer Genome Atlas (TCGA). *p < 0.05, **p < 0.01, ***p < 0.001. **B** *BCAM* mRNA expression in tumor and normal tissues from ccRCC, pRCC and chRCC obtained from 6 external datasets, including Higgins Renal, Gumz Renal, Beroukhim Renal, Yusenko Renal, Lenburg Renal and Jones Renal. **C** *BCAM* mRNA expression in tumor and normal tissues from ccRCC obtained from Gene Expression Omnibus (GEO) database, including GSE53757, GSE40435 and GSE66272. ****p < 0.0001. **D** Representative microphotographs of *BCAM* immunohistochemical staining in normal kidney tissue and ccRCC tissue by IHC. **E** *BCAM* mRNA expression in the single-cell-type clusters identified in normal kidney tissues. **F** *BCAM* protein expression in tumor and adjacent normal tissues from 6 patients in West China Hospital cohort by immunoblotting. The normalized data after quantitative analysis were shown on the right. **p < 0.01. **G** *BCAM* protein expression in tumor and normal tissues from ccRCC data of Clinical Proteomic Tumor Analysis Consortium (CPTAC). ***p < 0.001

Firstly, we evaluated the differential expression of *BCAM* in tumor and normal tissues at pan-cancer RNA level from TCGA (The Cancer Genome Atlas) database (Fig. 1A). Compared with the median expression level of corresponding normal tissues, *BCAM* expression remarkably decreased in all three classic subtypes of RCC (low-expressed proportions: ccRCC: 518/530, 97.74%; pRCC, 285/288, 98.96%; chRCC, 59/65, 90.77%; *p* < 0.001). The low expression of *BCAM* in RCC was sequentially validated in the other six external datasets (Fig. 1B). Moreover, its expression profile in ccRCC tumor samples was further verified from GSE53757 (*n* = 72), GSE40435 (*n* = 101) and GSE66272 (*n* = 26) datasets, all with a *p* < 0.001 (Fig. 1C). The results mentioned above confirmed that *BCAM* expression at mRNA level was lower than normal tissues in all the three subtypes of RCC (*p* < 0.05).

We then investigated the expression pattern of *BCAM* in RCC and adjacent normal tissues at the protein level using paraffin-embedded samples from HPA (The Human Protein Atlas) database. Firstly, immunohistochemical staining showed that for the *BCAM* protein in 12 ccRCC tumor tissues and 3 normal renal tissues, *BCAM* protein could be detected in all three normal tissues with moderate intensity and 75–25% quantity in membrane and cytoplasm of renal tubules, but was almost (11 of 12) undetectable in ccRCC tumor tissues (Fig. 1D). Using single-cell RNA sequencing (scRNA-seq) data of human kidney from GSE131685, a total of 25,279 cells from kidney tissues were divided into 14 clusters, including proximal tubular cells (c-0, c-1, c-2, c-3, c-4, c-5, c-6, c-8, c-10), distal tubular cells (c-11), collecting duct cells (c-12), T cells (c-7) and macrophages (c-8), B cells (c-13) [20]. Markers for collecting duct cells were AQP2, CLDN8, PVALB and TMEM213. Markers for distal tubular cells were SLC12A1, TMEM72 and UMOD. Markers for proximal tubular cells were MIOX, SLC22A8 and TMEM174. Other markers are listed in Additional file 1: Table S1. We identified that *BCAM* mRNA was mainly expressed in epithelial cell clusters (Fig. 1E). The expression level of *BCAM* mRNA was the highest in collecting duct cells c-12, followed by proximal tubular cells c-8 and c-5, which was consistent with the result that *BCAM* protein was mainly distributed in renal tubules of normal tissues according to IHC staining. Subsequently, we conducted western blotting with frozen samples in our center. The normalized results were consistent with the previous clues, suggesting the downregulation of *BCAM* protein in ccRCC (Fig. 1F). Finally, we utilized CPTAC (Clinical Proteomic Tumor Analysis Consortium) database to reconfirm the expression difference of *BCAM* between tumor and normal tissues in ccRCC at the protein level (Fig. 1G). A total of 110 primary tumor tissue samples were included and 84 normal tissue samples as the negative control. The result showed that 90.91% (100/110) of ccRCC tissues had lower protein expression of *BCAM* (below the median *BCAM* expression level of normal kidney tissue), and the difference in *BCAM* protein level between ccRCC and normal tissues was statistically significant (*p* < 0.001, fold change = 2). The expression of *BCAM* at protein level was consistent with those at RNA level, revealing that *BCAM* expression was indeed downregulated in renal tumor tissues at both mRNA and protein levels.

### Low BCAM expression was associated with adverse clinicopathological parameters and poor prognosis

**Fig. 2:**
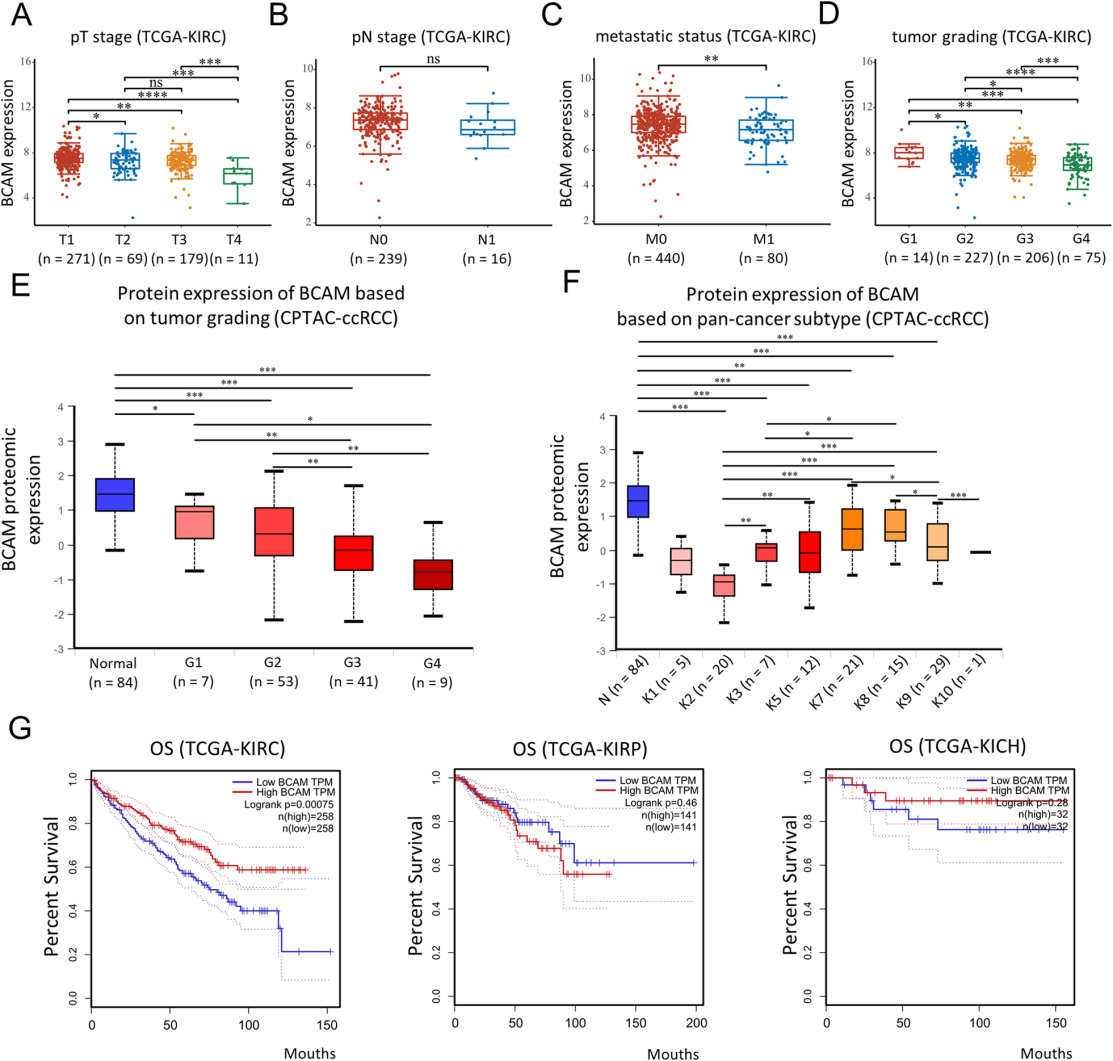
Low *BCAM* expression was associated with several clinicopathological characteristics and poor prognosis in ccRCC. **A** *BCAM* mRNA expression was associated with pT stage in ccRCC. **B** *BCAM* mRNA expression was not associated with pN stage in ccRCC. **C** *BCAM* mRNA expression was associated with metastatic status in ccRCC. **D** *BCAM* mRNA expression was associated with tumor grading in ccRCC. **E** *BCAM* protein expression was associated with tumor grading in ccRCC. **F** *BCAM* protein expression was associated with pan-cancer subtype in ccRCC. *p < 0.05, **p < 0.01, ***p < 0.001, ****p < 0.0001. **G** Kaplan–Meier analysis of the association between *BCAM* expression and OS in ccRCC, pRCC, chRCC

We then thoroughly investigated the potential functional roles of *BCAM* dysregulation in RCC. Clinicopathological parameters were collected and analyzed, including age, gender, pT stage, pN stage, metastatic status and tumor grading (ISUP grading). Firstly, we explored the relationship between *BCAM* mRNA expression and clinicopathological characteristics in the whole RCC cohort (Additional file 2: Table S2). The results revealed that *BCAM* mRNA expression negatively correlated with age, pT stage, metastatic status and tumor grade (all *p* < 0.05). The association of *BCAM* expression at the mRNA level with clinicopathological parameters was further analyzed and stratified by RCC histological types. For ccRCC, the expression of *BCAM* was again inversely correlated with pT stage, metastatic status and tumor grade (Fig. 2A–D). For pRCC and chRCC, there was no relationship between *BCAM* and clinicopathological variables except for the pN stage (Additional file 3: Fig. S1A–E). Finally, the association of *BCAM* mRNA expression from the TCGA database with overall survival (OS) was also analyzed, and the result demonstrated that low expression of *BCAM* in ccRCC was negatively related to OS, while within pRCC and chRCC, *BCAM* expression did not affect survival status (Fig. 2G).

After that, we analyzed data from the CPTAC database and found that the relationship between *BCAM* expression at the protein level and tumor grading was consistent with that at the mRNA level (Fig. 2E). Moreover, according to mass-spectrometry-based proteomic classification (K1–K10), 194 ccRCC enrolled in the database could be subclassified into 8 subgroups (except K4 and K6) [21]. Each subgroup had notable features. For example, K2 and K3 were featured by the activation of immunity signaling pathways. In general, *BCAM* expression level among different proteomic subtypes was remarkably lower than that in adjacent normal tissues, and it was noted that those who belonged to K2 subtype, which was associated with adaptive immune response and T cell activation, had the lowest expression level of *BCAM*, indicating the potential association between *BCAM* downregulation and the activation of immune-related signatures (Fig. 2F).

### Potential mechanisms of BCAM dysregulation in ccRCC

#### Genetic analysis indicated that DNA mutation and CNV had no certain effects on *BCAM* downregulation

**Fig. 3:**
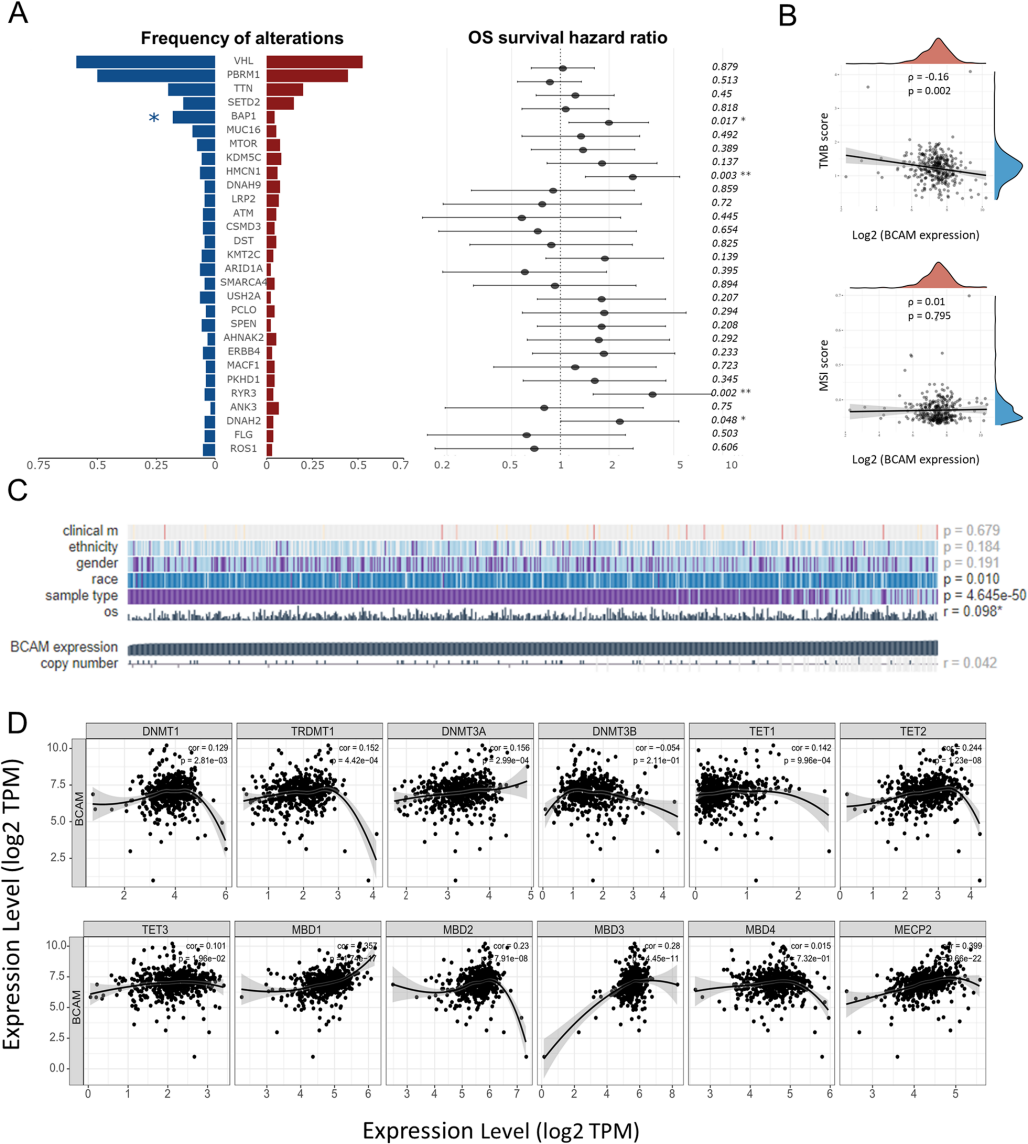
*BCAM* expression was associated with gene mutation features and DNA methylation modification in ccRCC. **A** Alteration frequency of the genes with the highest alteration frequency in the whole ccRCC cohort between the *BCAM*-low and BCAM-high subgroups, and the correlations with these genes and the OS prognosis of ccRCC patients. **B** The association between *BCAM* expression and tumor mutational burden (TMB) and microsatellite variation (MSI). **C** The association between *BCAM* expression and copy number variation (CNV). **D** The correlation of *BCAM* expression and the expression of DNA methylation-related genes

Since the expression of *BCAM* was correlated with various clinical characteristics and predicted the prognosis in ccRCC, it might be of clinical significance to divide ccRCC samples into *BCAM*-low and *BCAM*-high subgroups. We then set the median expression of *BCAM* as the cutoff value and further seek for multi-omics differences between the *BCAM*-high and *BCAM*-low subgroups. To find out the potential regulatory mechanism of the *BCAM* gene in ccRCC, we initially detected the DNA alteration and copy number variation (CNV) status, based on the differential expression of *BCAM* within ccRCC data in the TCGA cohort. The extremely low frequency (0.3%) of somatic mutation with *BCAM* itself did not explain its high percentage of low expression among ccRCC (Additional file 3: Fig. S1F). As shown in Fig. 3A, a much higher frequency of BAP1 mutation was the only alteration related to *BCAM* low expression, predicting the poor prognosis (17.24% vs 4.47%, *p* < 0.05). In *BCAM*-low subgroup, patients with BAP1 mutation had a worse OS than those without BAP1 mutation (Additional file 3: Fig. S1G).

We also analyzed the correlation between *BCAM* gene expression and tumor mutation burden (TMB)/microsatellite instability (MSI). In ccRCC, lower expression of *BCAM* was correlated with higher TMB (Fig. 3B). At the same time, there was no relation between *BCAM* and MSI status. In the end, we investigated the association of CNV with *BCAM* expression. We found that there was no correlation (r = 0.042) (Fig. 3C). Taken together, these results suggested that genetic alteration could not explain the dysregulation of *BCAM*; instead, low *BCAM* expression could be associated with a higher frequency of BAP1 mutation in ccRCC.

#### Hypermethylation was closely related to *BCAM* downregulation

**Fig. 4:**
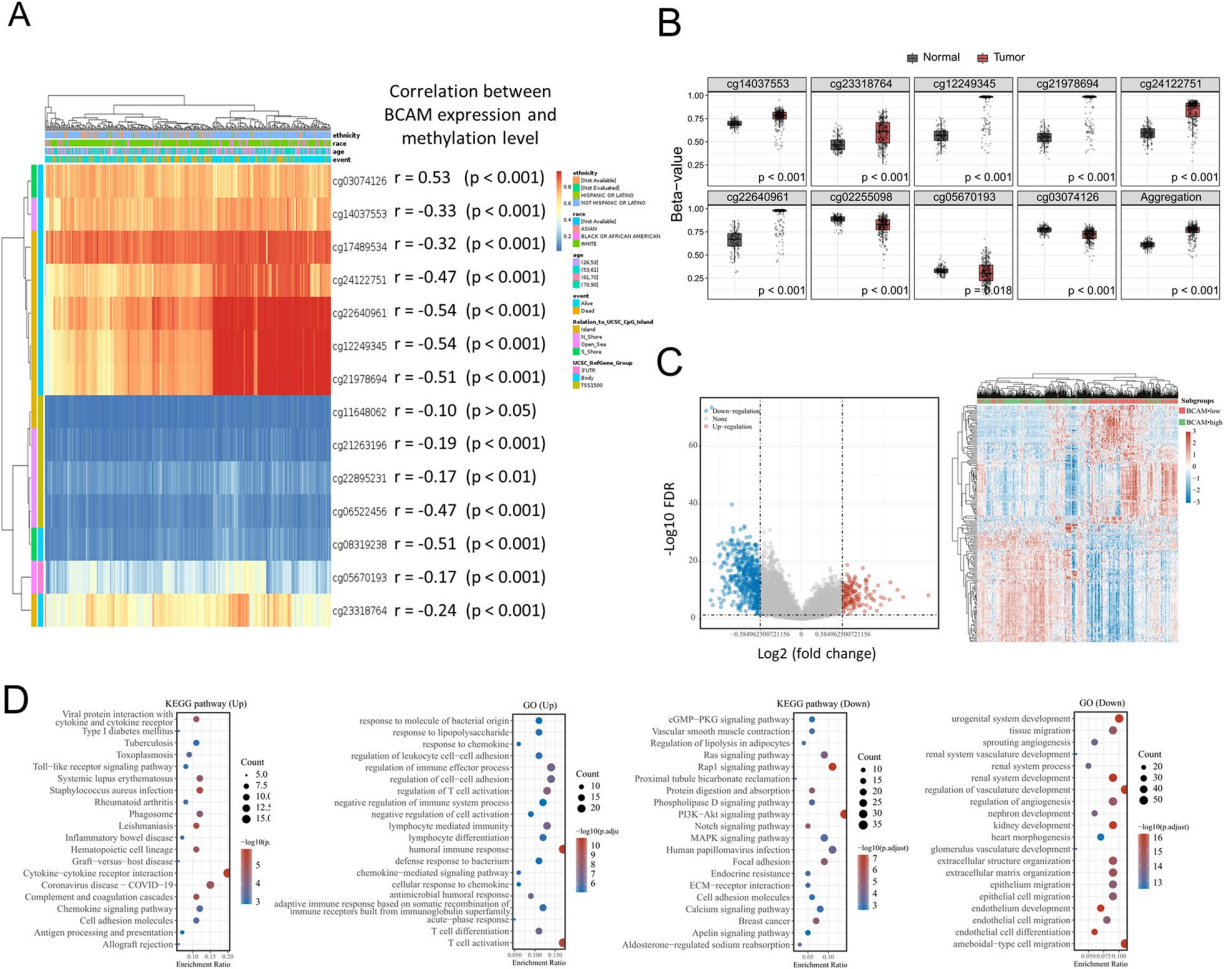
*BCAM* expression was associated with DNA methylation status in ccRCC, and the *BCAM*-low and *BCAM*-high subgroups had different enriched functions and pathways. **A** The association of methylation level with gene subregions. **B** The DNA methylation level of different probes between normal and tumor tissues. **C** Differential genes between the *BCAM*-low and *BCAM*-high subgroups and 50 upregulated genes and 50 downregulated genes with the largest differential changes. **D** KEGG pathway enrichment analysis and GO enrichment analysis of genes upregulated in the *BCAM*-low subgroup and genes upregulated in the *BCAM*-high subgroup

Next, we explored whether the downregulation of *BCAM* in ccRCC might be regulated by epigenetic modification using the TCGA database. We initially took the DNA methylation modification into account and exhibited the correlation between *BCAM* gene and several genes involved in DNA methylation (Fig. 3D). As the results illustrated, *BCAM* gene positively correlated with the expression of most methylation related enzymes in ccRCC, such as MECP2 (r = 0.399, *p* < 0.001), MBD3 (r = 0.28, *p* < 0.001) and TET2 (r = 0.244, *p* < 0.001). We then generated a waterfall plot to associate methylation levels with gene subregions visually based on the sequencing results of 450 k DNA methylation chips in the TCGA project (Fig. 4A). Several *BCAM*-related probes had a high extent of methylation, including cg03074126, cg14037553, cg17489534, cg24122751, cg22640961, cg12249345, cg21978694, cg05670193 and cg23318764. We further thoroughly investigated the specific correlations between the methylation level of these probes and *BCAM* expression in ccRCC. The results revealed that, in the 333 samples of ccRCC, the methylation level of 8 out of 9 probes negatively correlated with *BCAM* gene expression, in which CpG island-related probe cg22640961 (r = − 0.54, *p* < 0.001) showed the strongest negative correlation, followed by cg12249345 (r = − 0.52, *p* < 0.001), cg21978694 (r = − 0.51, *p* < 0.01), cg24122751 (r = − 0.47, *p* < 0.001), cg14037553 (r = − 0.33, *p* < 0.001), cg17489534 (r = − 0.32, *p* < 0.001), cg23318764 (r = − 0.24, *p* < 0.001) and cg05670193 (r = − 0.17, *p* < 0.01). However, cg03074126 (r = 0.53, *p* < 0.001) was the exception and its methylation level strongly positively correlated with *BCAM* expression. Also, when integrating the results of CpG island-related probes, the aggregation result showed a correlation coefficient of − 0.54 (*p* < 0.001), indicating that the low expression of *BCAM* could be attributed to its CpG island-related methylation modification. Furthermore, we studied whether the methylation degree of these probes differed between tumor and normal tissues. We found that all the methylation level of probes, except for cg03074126 and cg05670193, was significantly higher in tumor tissues than that in normal tissues in ccRCC (Fig. 4B). Given the above-analyzed results that low *BCAM* expression coexisted with a higher frequency of BAP1 mutation in ccRCC, we further tried to explore the relationship between BAP1 mutation status and *BCAM* methylation level. To our surprise, three probes of *BCAM*, including cg06522456 (*p* < 0.01), cg08319238 (*p* < 0.001) and cg14037553 (*p* < 0.01), showed higher level of methylation in ccRCC with BAP1 mutation compared to BAP1 wild-type ccRCC, indicating the potential mechanism of BAP1 mutation regulating *BCAM* expression level (Additional file 3: Fig. S1H).

In general, these results indicated that *BCAM*-related epigenetic modification was obviously associated with its gene regulation and RCC patient prognosis.

### Different expressions of BCAM indicated different transcriptomic characteristics

We attempted to further explore the transcriptomic differences between *BCAM*-low and *BCAM*-high groups and find clues for potential treatment schemes, respectively. Therefore, we first observed the differentially expressed genes between the *BCAM*-low and *BCAM*-high subgroups based on the TCGA database (Fig. 4C). The results demonstrated that, compared to *BCAM*-high subgroup, there were 145 up-expressed genes and 539 down-expressed genes in *BCAM*-low subgroup (Additional file 4: Table S3).

#### Low *BCAM* expression was associated with high immunogenicity

**Fig. 5:**
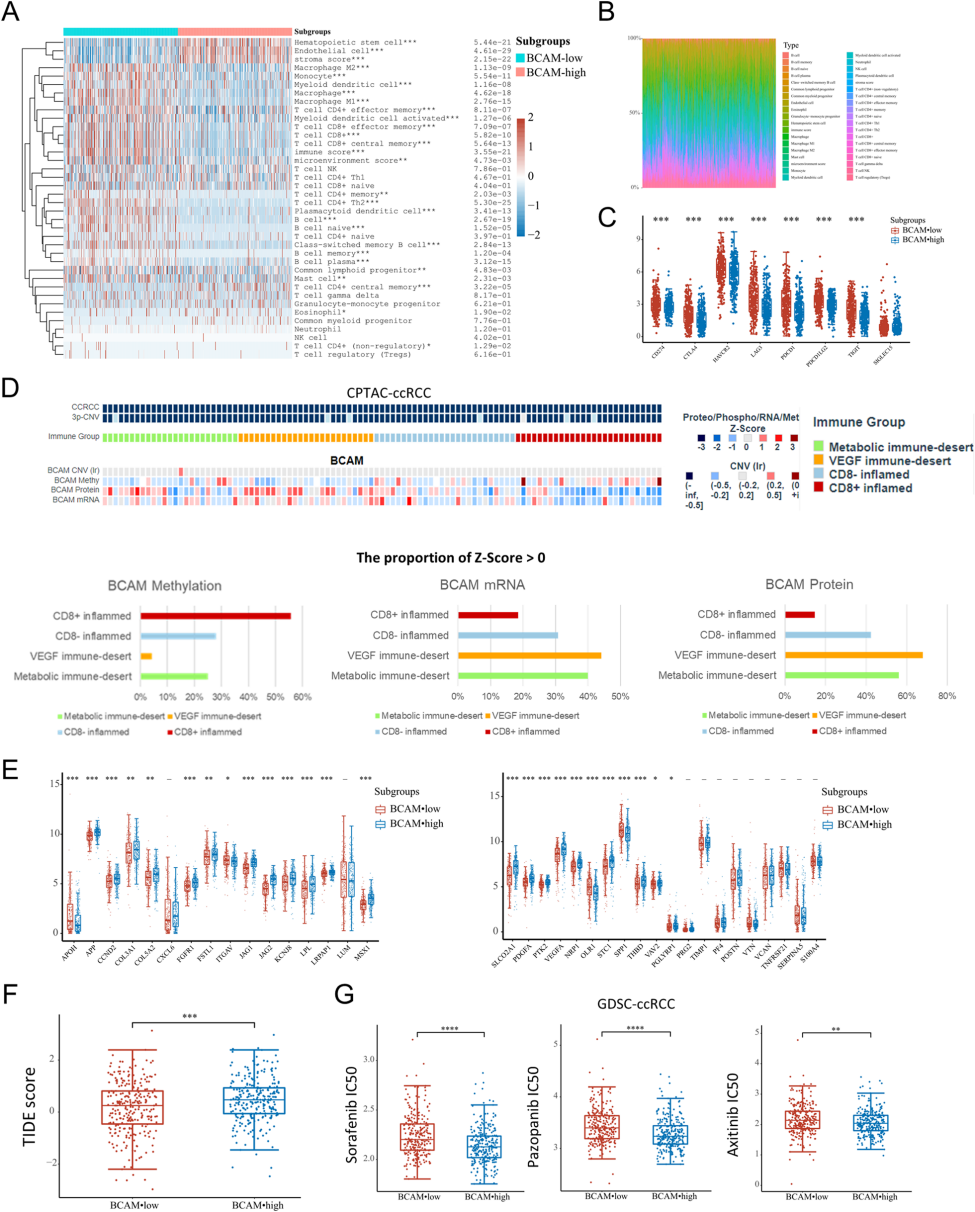
Low *BCAM* expression was associated with high immunogenicity; distinct *BCAM* expression patterns could indicate potential therapeutic strategies in ccRCC. **A** Immune cell score in the *BCAM*-low and *BCAM*-high subgroups. *p < 0.05, **p < 0.01, ***p < 0.001. **B** The percentage abundance of tumor-infiltrating immune cells in each sample. **C** The expression of immune checkpoints in the *BCAM*-low and *BCAM*-high subgroups. ***p < 0.001. **D** The association between different immune subgroups and the mRNA and protein expression and methylation level of *BCAM*, and the proportion of Z-Score > 0 of the 4 immune subgroups in *BCAM* methylation level, mRNA expression level and protein expression level, respectively. **E** The expression of angiogenesis-related genes in the *BCAM*-low and *BCAM*-high subgroups. *p < 0.05, **p < 0.01, ***p < 0.001. **F** Tumor Immune Dysfunction and Exclusion (TIDE) score of the *BCAM*-low and *BCAM*-high subgroups. **G** Distribution of Sorafenib, Pazopanib and Axitinib IC50 scores in the *BCAM*-low and *BCAM*-high subgroups. *p < 0.05, **p < 0.01, ***p < 0.001, ****p < 0.0001

Both the KEGG pathway (Up) enrichment analysis and the GO (Up) enrichment analysis demonstrated that the up-expressed genes among the *BCAM*-low subgroup were mostly enriched in the immune-related signaling pathway, including “cytokine–cytokine receptor interaction,” “chemokine signaling pathway,” “cell adhesion molecules” and “antigen processing and presentation” (Fig. 4D). To further investigate the immune status in ccRCC, we compared the immune infiltration between *BCAM*-low and *BCAM*-high subgroups. After mining data from TCGA cohort, we found that in ccRCC samples, most immune cell types were increased in *BCAM*-low subgroup, such as M2 macrophage (*p* < 0.001), monocyte (*p* < 0.001), myeloid dendritic cell (*p* < 0.001), CD8 + T cells (*p* < 0.001) and CD4 + Th2 cells (*p* < 0.001) (Fig. 5A). The composition of these immune cell subpopulations in tumor microenvironment (TME) of ccRCC was also calculated (Fig. 5B) [22]. Data from CPTAC database further demonstrated that, when compared with metabolic immune desert, VEGF immune desert and CD8− inflamed subgroups, the CD8 + inflamed subgroup had a lowest *BCAM* mRNA (the proportion of Z-Score > 0 = 18.5%) and protein (the proportion of Z-Score > 0 = 16.0%) expression level, and a highest *BCAM* methylation level (the proportion of Z-Score > 0 = 55.6%), consistent with the above results (Fig. 5D). Additionally, the VEGF immune desert subgroup had the highest *BCAM* mRNA (the proportion of Z-Score > 0 = 44.0%) and protein (the proportion of Z-Score > 0 = 68.0%) expression level and the lowest *BCAM* methylation level (the proportion of Z-Score > 0 = 4.2%) among the 4 immune subgroups. We also evaluated the expression of 8 immune checkpoints of *BCAM*-low and *BCAM*-high subgroups using the TCGA database to see whether there were differences in expression level in ccRCC (Fig. 5C). The results revealed that, compared to *BCAM*-high subgroup, the expression of CD274 (*p* < 0.001), CTLA4 (*p* < 0.001), HAVCR2 (*p* < 0.001), LAG3 (*p* < 0.001), PDCD1 (*p* < 0.001), PDCD1LG2 (*p* < 0.001) and TIGIT (*p* < 0.001) was elevated by varying degrees in *BCAM*-low subgroup. Further analysis revealed that these 7 immune checkpoints all had lower methylation levels in *BCAM*-low subgroup compared to the *BCAM*-high subgroup (*p* < 0.01) (Additional file 5: Fig. S2A). The results demonstrated that immune checkpoints might lead to immune escape of tumor cells in ccRCC.

#### High *BCAM* expression was associated with the enrichment of angiogenesis

On the other hand, the KEGG and GO enrichment analysis for the *BCAM*-high subgroup showed that the angiogenesis-related pathways were remarkably enriched (Fig. 4D). We then attempted to further confirm whether the extent of angiogenesis activation was higher in the *BCAM*-high subgroup of ccRCC sample. The expression level of 36 related genes between *BCAM*-low subgroup and *BCAM*-high subgroup was compared, and the results illustrated that 16 angiogenesis related genes, including JAG2, JAG1, MSX1, SLCO2A1, APP, PDGFA, PTK2, VEGFA, NRP1, STC1, FGFR1, LPL, KCNJ8, LRPAP1, CCND2 and THBD, were significantly upregulated in *BCAM*-high subgroup (*p* < 0.001) (Fig. 5E), and only four were upregulated among BCAM-low subgroup. We also found that for JAG1, MSX1, SLCO2A1, APP, PTK2, NRP1, STC1, FGFR1, KCNJ8, LRPAP1, CCND2 and THBD, the methylation degree in *BCAM*-high subgroup was lower than that in *BCAM*-low subgroup (*p* < 0.05) (Additional file 5: Fig. S2B). All the findings revealed that more angiogenesis related genes upregulation was enriched in *BCAM*-high ccRCC patients.

These transcriptomic results suggested that immune-related signaling pathways were overactivated in the *BCAM*-low subgroup. In contrast, more angiogenesis-related signaling pathways were activated in the *BCAM*-high subgroup, which implied the different therapeutic strategies in practice.

### Potential therapeutic strategies based on distinct BCAM expression patterns in ccRCC

Finally, we looked at the predictive value of *BCAM* in the treatment of ccRCC. The sensitivity of tyrosine kinase inhibitor (TKI) drugs was calculated based on the GSDC database, comparing within differential *BCAM* expression subgroups. The results showed that the half-maximal inhibitory concentration (IC50) of Sorafenib (*p* < 0.0001), Pazopanib (*p* < 0.0001) and Axitinib (*p* < 0.01) among the *BCAM*-high subgroup was significantly lower than those within *BCAM*-low group (Fig. 5F). These results indicated that anti-angiogenesis therapy should be considered for ccRCC patients with *BCAM*-high expression.

Tumor Immune Dysfunction and Exclusion (TIDE) algorithm modeled immune evasion in tumors by combining both T cell dysfunction and exclusion signatures. The higher the TIDE prediction score was, the worse the immune checkpoint inhibitor (ICI) response was. Using the TCGA database, we calculated and compared the TIDE scores of the *BCAM*-low and *BCAM*-high subgroups. In contrast with the *BCAM*-high subgroup, the TIDE score of the *BCAM*-low subgroup was much lower (*p* = 0.00052) (Fig. 5G), indicating that the *BCAM*-low subgroup might respond better to ICI-based immunotherapy. Considering the upregulation of multiple different immune checkpoints, including CD274, CTLA4, HAVCR2, LAG3, PDCD1, PDCD1LG2 and TIGIT, double or triple ICIs combination therapy might be promising among ccRCC with *BCAM*-low expression.

## Discussion

In this study, we found that *BCAM* was abnormally downregulated in all three classic types of RCC. In ccRCC, the low expression of *BCAM* was associated with adverse clinicopathological parameters and poor prognosis. Although genetic analysis could not explain the dysregulation of *BCAM* in ccRCC, the high frequency of BAP1 mutation and higher TMB among the *BCAM*-low subgroup need more attention. DNA methylation modification was at least partially attributed to the *BCAM* downregulation in ccRCC. KEGG/GO enrichment analysis and TIDE score evaluation revealed much higher immunogenicity within *BCAM*-low subgroup, strongly indicating the potential and promising efficacy of ICI-based immunotherapy. On the contrary, classic anti-angiogenetic therapy should be well considered among patients with increased expression of *BCAM*.

*BCAM* expression profiles have been studied in various solid tumors. A majority of studies identified the high expression of *BCAM* among malignancies, including epithelial skin tumor, ovarian cancer, bladder cancer and gastric cancer [12–15]. The low expression of *BCAM* has only been found in thyroid cancer and colon cancer [16, 17]. Previous studies identified that the dysfunction of *BCAM* might be involved in cell adhesion, migration and tumor metastasis, which might explain the association of adverse clinicopathological parameters and poor prognosis with low expression of *BCAM* in ccRCC.

The heterogeneity of *BCAM* expression among different solid tumors determined the differential regulation mechanism of *BCAM* in different types of cancer. Unfortunately, only one study tried to explore the probable regulation of *BCAM* in gastric cancer [15]. Multiple pathways can lead to gene dysregulation, among which epigenetic modification is indispensable. DNA methylation, another crucial epigenetic modification of the genome, is closely related to tumorigenesis [23]. In our present study, we found that in ccRCC, some DNA methylation status, including hypermethylation of cg14037553, cg17489534, cg24122751, cg22640961, cg12249345, cg21978694 and cg23318764, could explain the downregulation of *BCAM*. Although DNA mutation and CNV burden did not affect the dysregulation of *BCAM*, the genetic analysis found *BCAM* low expression had a closer association with higher TMB and high frequency of BAP1 mutation. TMB has been confirmed to be associated with poorer prognosis, advanced pathological stages and higher tumor grades, consistent with our results [24]. BAP1 is a tumor suppressor gene inactivated in 15% of ccRCC [25]. Loss-of-function BAP1 mutations are associated with higher tumor grade and poorer prognosis in ccRCC [26]. Previous research in uveal melanoma demonstrated that BAP1 could regulate the expression of multiple cell adhesion molecules [27]. As one adhesion molecule, *BCAM* may also be regulated by BAP1 in ccRCC, although there is no evidence yet. Another research in uveal melanoma revealed that BAP1 could induce methylomic repatterning [28]. According to our results, the expression of *BCAM* might be regulated mainly by DNA methylation, and we further discovered a higher methylation level of BCAM in ccRCC with BAP1 mutation. All these findings indicate that BAP1 may interact with *BCAM* and epigenetically silence the expression of *BCAM*, which should be further studied and confirmed. Additionally, survival analysis showed that patients with *BCAM* low expression concomitant with BAP1 mutation had a worse prognosis, reconfirming the adverse impact of BAP1 mutation among ccRCC.

Currently, anti-angiogenetic therapy and ICI-based immunotherapy are the mainstay treatments for ccRCC. Both these therapies may have specific applicable populations, and relevant biomarkers have been explored to distinguish responses to these two therapies [29–31]. However, due to low potency and low practicability, no clinically implemented biomarkers have been approved for rational selection of therapies. We are interested in the distribution of angiogenesis- and immune-related signaling pathways between *BCAM*-low and *BCAM*-high subgroups within ccRCC. Intriguingly, the transcriptomic analysis demonstrated the high immunogenicity of the BCAM-low subgroup and high angiogenesis of the *BCAM*-high subgroup. For the *BCAM*-low subgroup, pathways including “cytokine-cytokine receptor interaction,” “chemokine signaling pathway,” “cell adhesion molecules” and “antigen processing and presentation” were mostly enriched, which was a vital link of immune response and could potentially induce cancer [32, 33]. And for the *BCAM*-high subgroup, angiogenesis-related pathways, including “regulation of angiogenesis” and “sprouting angiogenesis.” Subsequent analysis confirmed that we could make the therapeutic decision by using *BCAM* expression status for patients with ccRCC in practice. That is to say, for patients with *BCAM*-high expression, anti-angiogenetic therapies should be the optimal stand of care, while for patients with *BCAM*-low expression, ICI-based immunotherapy should be of priority. Interestingly, the immune phenotypes of TME could be used to forecast the efficacy of different therapeutic strategies. The CD8 + inflamed subgroup was considered to benefit the most from immunotherapy, and VEGF immune desert subgroup might benefit from anti-angiogenesis therapy, instead of immunotherapy [22]. Our results demonstrated that patients with *BCAM*-low expression were mainly distributed in CD8 + inflamed subgroup, while patients in VEGF immune desert subgroup showed the highest *BCAM* expression. In addition, within the *BCAM*-low subgroup, 17.24% of patients were found to coexist with BAP1 mutation. Whether BAP1 mutation could interfere with immunotherapy efficacy needs further investigation. Also, except for CD274 overexpression, other immune checkpoints, such as CTLA4, HAVCR2, LAG3, PDCD1, PDCD1LG2 and TIGIT, were overexpressed in ccRCC with *BCAM*-low expression. Combining a set of immune checkpoint biomarkers is essential to predict the efficacy of ICI-based immunotherapy and, eventually, help make decisions if double or triple immunotherapy is necessary.

## Conclusions

This study reveals the expression profile of *BCAM* in RCC and found that the downregulation of *BCAM* is significantly associated with a poorer prognosis of ccRCC. DNA methylation modification might be one of the leading causes of *BCAM* dysregulation. The *BCAM*-low subgroup characterized by high immune infiltration and immune checkpoint expression may determine the more favorable response to ICI-based immunotherapies. At the same time, anti-angiogenetic therapies may be more suitable for the *BCAM*-high subgroup characterized by enrichment of angiogenesis. Our data indicate that the expression of *BCAM* can predict the prognosis of ccRCC and could suggest potential therapeutic strategies in ccRCC based on different molecular characterizations.

## Methods

### RNA expression analysis

First, we evaluated the diverse expression level of *BCAM* gene in tumor tissues and adjacent normal tissues in varieties of cancers based on TCGA (The Cancer Genome Atlas) database and the results were displayed through TIMER (Tumor Immune Estimation Resource) [34, 35]. In this cohort, 533 tumor tissue samples and 72 adjacent normal tissue samples for kidney renal clear cell carcinoma (ccRCC), 290 tumor tissue samples and 32 adjacent normal tissue samples for kidney renal papillary cell carcinoma (pRCC), 66 tumor tissue samples and 25 adjacent normal tissue samples for kidney chromophobe (chRCC) were analyzed for RNA expression pattern. The proportion of *BCAM* expression below the median *BCAM* expression level of normal kidney tissue at RNA level in ccRCC, pRCC and chRCC was further calculated. Then, to validate the findings from the TCGA database, we also pooled a total of 6 external datasets with 311 cases enrolled, including Higgins Renal (*n* = 44), Gumz Renal (*n* = 20), Beroukhim Renal (*n* = 70), Yusenko Renal (*n* = 67), Lenburg Renal (*n* = 18) and Jones Renal (*n* = 92), and performed Student’s t test to determine the expression difference of *BCAM* gene between ccRCC, pRCC, chRCC and control samples, respectively, using Oncomine platform [36–42]. Genes that reached a *p* value of 0.05, fold change of 1.5, and ranked the top 10% were considered as differentially expressed. In addition, 3 microarray datasets from GEO (Gene Expression Omnibus) database were downloaded and used to verify the expression level of *BCAM* in ccRCC, including GSE53757, GSE40435 and GSE66272, all containing no less than 20 human ccRCC and adjacent normal tissues [43–47]. Wilcox rank sum test was selected as the significance *p* value test method. If *p* < 0.05, we concluded that the results were statistically significant. The data of single-cell transcriptome profiling for healthy human kidney tissue were retrieved from GSE131685 [20]. Scanpy package in Python 3.8.5 was used for downstream analysis. In total, 25,279 cells from the kidneys of three human donors were included in the analysis after filtering out cells of poor quality. Subsequently, the cell counts were normalized to have a total count per cell of 10,000. The valid cells were then clustered using the Louvain clustering function within single-cell analysis in Python. Additionally, the features of cells were projected into a principal component analysis (PCA) space with 50 components using UMAP, and a k-nearest neighbors graph was generated. The resolution of clustering was set as 1.0. The *BCAM* mRNA expression in each cell type cluster was shown by the bar chart.

### Protein expression analysis

Moreover, we verified the *BCAM* expression at protein level based on the immunohistochemical results of 12 cases of tumor tissue samples and 3 normal tissue samples through HPA [48]. Antibody used for staining was HPA005654, provided by Atlas Antibodies, and its working concentration was 0.0975 mg/ml. Then, we conducted a Western blot analysis to further investigate the difference of *BCAM* protein expression between RCC tissues and adjacent normal tissues in our patient cohort. Total protein from tumor samples and corresponding normal tissues was isolated using RIPA Lysis Buffer, and protein concentration was measured by BCA protein assay method. The primary antibody (anti-*BCAM*, sc-365191, Santa Cruz; anti-GAPDH, #5174, Cell Signaling Technology) was incubated overnight at a dilution rate of 1:1000. Western blot analysis of *BCAM* and GAPDH was according to standard protocols. Finally, we utilized data from CPTAC to furtherly validate *BCAM* proteomic expression difference between tumor and normal tissues using UALCAN tool and the proportion of *BCAM* expression below the median *BCAM* expression level of normal kidney tissue at protein level in ccRCC was also calculated [49]. We further applied one proteome-based classification method based on mass-spectrometry-based proteomic profiling of 532 cancers representing six tissue-based types (breast, colon, ovarian, renal and uterine) to classify ccRCC from the CPTAC database [21]. This method had the potential to identify molecular subtypes and associated pathways characteristics that might be otherwise missed using transcriptomics. Ten pan-cancer subtypes were differentiated and described. K1 was related to overexpression of proteasome complex proteins, glycolysis proteins and pentose phosphate pathway proteins. K2 was associated with adaptive immune response and T cell activation. K3 was associated with an innate immune response. K4 only represented basal-like breast cancer. K5 was marked by an epithelial signature. K6 and K7 were both stromal-related. K8 was featured by overexpression of Golgi apparatus-related proteins. K9 was only found in ccRCC. K10 was associated with overexpression of endoplasmic reticulum-related proteins.

### Genetic alteration analysis

We then focused our research on ccRCC entirely in view of the analysis results above. First, we explored the alteration frequency of the *BCAM* gene among genetic mutation data of ccRCC based on the TCGA cohort. Furthermore, in order to identify the somatic landscape of ccRCC in the TCGA cohort, we selected genes with the highest alteration frequency in the whole ccRCC cohort and compared whether there existed a difference in the frequency of alterations of these genes between the *BCAM*-low and *BCAM*-high subgroups. And the correlations between these genes and the OS survival of ccRCC patients were also analyzed. Moreover, the “ggstatsplot” package was used to analyze comprised RNA-seq data of 530 ccRCC samples from the TCGA project and the correlation of *BCAM* gene expression and TMB/MSI was described using Spearman rank analysis. A *p* value of less than 0.05 was considered statistically significant. Ultimately, the correlation between CNV and expression level of *BCAM* and the CNV types of *BCAM* gene was investigated by developing a MEXPRESS plot [50].

### Epigenetic modification analysis

Firstly, we explored the correlation between the *BCAM* gene and several genes which were involved in DNA methylation, including DNMT1, TRDMT1, DNMT3A, DNMT3B, TET1, TET2,TET3, MBD1, MBD2, MBD3, MBD4 and MECP2 [51]. At the same time, we adjusted the association by tumor purity. Spearman rank test was applied to verify the correlation and the purity-adjusted partial spearman’s rho value as the degree of correlation was exhibited as heatmap, and *p* < 0.05 was considered statistically significant. Then we overviewed the methylation status of different probes in the *BCAM* DNA in the TCGA ccRCC cohort based on MethSurv [52]. The specific correlations between methylation level and *BCAM* expression in ccRCC were analyzed through SMART [53]. We selected beta value for analysis and Pearson method for calculating the correlation coefficient. Next, we focused on the probes which had a high degree of methylation, including cg03074126, cg14037553, cg17489534, cg24122751, cg22640961, cg12249345, cg21978694, cg05670193 and cg23318764, and investigated the difference of methylation degree of these probes between tumor and normal tissues in ccRCC. Ultimately, the correlation between BAP1 mutation status and *BCAM* methylation level was analyzed.

### Potential therapeutic strategies analysis

We first obtained the corresponding clinical information of RNA sequencing data from TCGA ccRCC cohort. Then we predicted the chemotherapeutic response for ccRCC samples based on the GDSC (Genomics of Drug Sensitivity in Cancer). TKI drugs, including Sunitinib, Sorafenib, Pazopanib and Axitinib, and evaluated the therapeutic response of the *BCAM*-low group and the *BCAM*-high group using IC50. Ridge regression was utilized to estimate each sample’s IC50. All parameters were set by default with the removal of the batch effect of combat and the tissue type, and the repeated gene expression was summarized as the mean value. Next, we predicted the potential immune checkpoint blockade response of the *BCAM*-low and *BCAM*-high subgroups with TIDE algorithm, a method to model two primary mechanisms of tumor immune evasion, in which the high TIDE score was associated with the poor efficacy of ICI therapy and short survival time after ICI treatment [54]. The results were demonstrated by the “ggplot2” and “ggpubr” package. Refer to Additional file 6 for methods of clinicopathology and prognosis analysis, functional and pathway enrichment analysis, immune infiltration and immune checkpoint analysis.

## Supplementary Information

**Additional file 1**: **Table S1**. Markers for different cell types in scRNA-seq.

**Additional file 2**: **Table S2**. Clinical characteristics of the BCAM-low and BCAM-high subgroups of RCC patients.

**Additional file 3**: **Fig. S1**. **A** BCAM mRNA expression was not associated with pT stage in pRCC. **B** BCAM mRNA expression was associated with pN stage in pRCC. **C** BCAM mRNA expression was not associated with metastatic status in pRCC. **D** BCAM mRNA expression was not associated with pT stage in chRCC. **E** BCAM mRNA expression was associated with pN stage in chRCC. *p < 0.05, **p < 0.01. **F** Mutation distribution and protein domains for BCAM gene in ccRCC with the labeled recurrent hotspots. **G** Kaplan-Meier analysis of the association between BAP1 mutation status and OS in the BCAM-low subgroup. **H** The methylation level of BCAM probes between ccRCC with different BAP1 mutation status. MT = mutation type, WT = wild type. **p < 0.01, ***p < 0.001.

**Additional file 4**: **Table S3**. Differential genes between the BCAM-low and BCAM-high subgroups.

**Additional file 5**: **Fig. S2**. **A** Methylation level of immune checkpoints with higher expression in the BCAM-low subgroup between the BCAM-low and BCAM-high subgroups. **B** Methylation level of angiogenesis-related genes which were significantly upregulated in the BCAM-high subgroup (*p* < 0.001) between the BCAM-low and BCAM-high subgroups. *p < 0.05, **p < 0.01, ***p < 0.001.

**Additional file 6**: Methods of clinicopathology and prognosis analysis, functional and pathway enrichment analysis, immune infiltration and immune checkpoint analysis.

## Data Availability

Transcriptomic data of ccRCC, pRCC and chRCC tissues and adjacent normal tissues were derived from the TCGA database and are freely available from https://portal.gdc.cancer.gov/. The 6 external datasets used for the first validation were obtained from teams of Prof. Brooks, Prof. Copland, Prof. Signoretti, Prof. Kovacs, Prof. Christman and Prof. Libermann [36–41]. The 3 ccRCC datasets used for the second validation were available from GEO http://www.ncbi.nlm.nih.gov/geo/ under Accession Numbers GSE53757, GSE40435 and GSE66272. Proteomic data of ccRCC tissues and adjacent normal tissues were obtained from the HPA database https://www.proteinatlas.org/, West China Hospital cohort and CPTAC database https://cptac-data-portal.georgetown.edu/. Clinicopathological data, prognostic information, genomic, methylomic and immunomic data were all derived from the TCGA database. Data of drug sensitivity were derived from the GDSC database https://www.cancerrxgene.org/.

## Abbreviations

RCC: Renal cell carcinoma
BCAM: Basal cell adhesion molecule
TIDE: Tumor immune dysfunction and exclusion
GDSC: Genomics of drug sensitivity in cancer
TME: Tumor microenvironment
ccRCC: Clear cell renal cell carcinoma
pRCC: Papillary renal cell carcinoma
chRCC: Chromophobe renal cell carcinoma
IgSF: Immunoglobulin superfamily
TCGA: The Cancer Genome Atlas
HPA: The Human Protein Atlas
scRNA-seq: Single-cell RNA sequencing
CPTAC: Clinical Proteomic Tumor Analysis Consortium
OS: Overall survival
CNV: Copy number variation
TMB: Tumor mutation burden
MSI: Microsatellite instability
TKI: Tyrosine kinase inhibitor
ICI: Immune checkpoint inhibitor
TIMER: Tumor immune estimation resource
GEO: Gene expression omnibus
PCA: Principal component analysis
IC50: Half-maximal inhibitory concentration
GEPIA: Gene Expression Profiling Interactive Analysis
GO: Gene Ontology
KEGG: Kyoto Encyclopedia of Gene and Genomes
