# Supplementary figures and images for: Integrated multi-omics analyses reveal that BCAM is associated with epigenetic modification and tumor microenvironment subtypes of clear cell renal cell carcinoma
Source: Clin Epigenetics. 2022 Aug 8;14:99. doi: 10.1186/s13148-022-01319-2 (PMC9361577; doi:10.1186/s13148-022-01319-2)

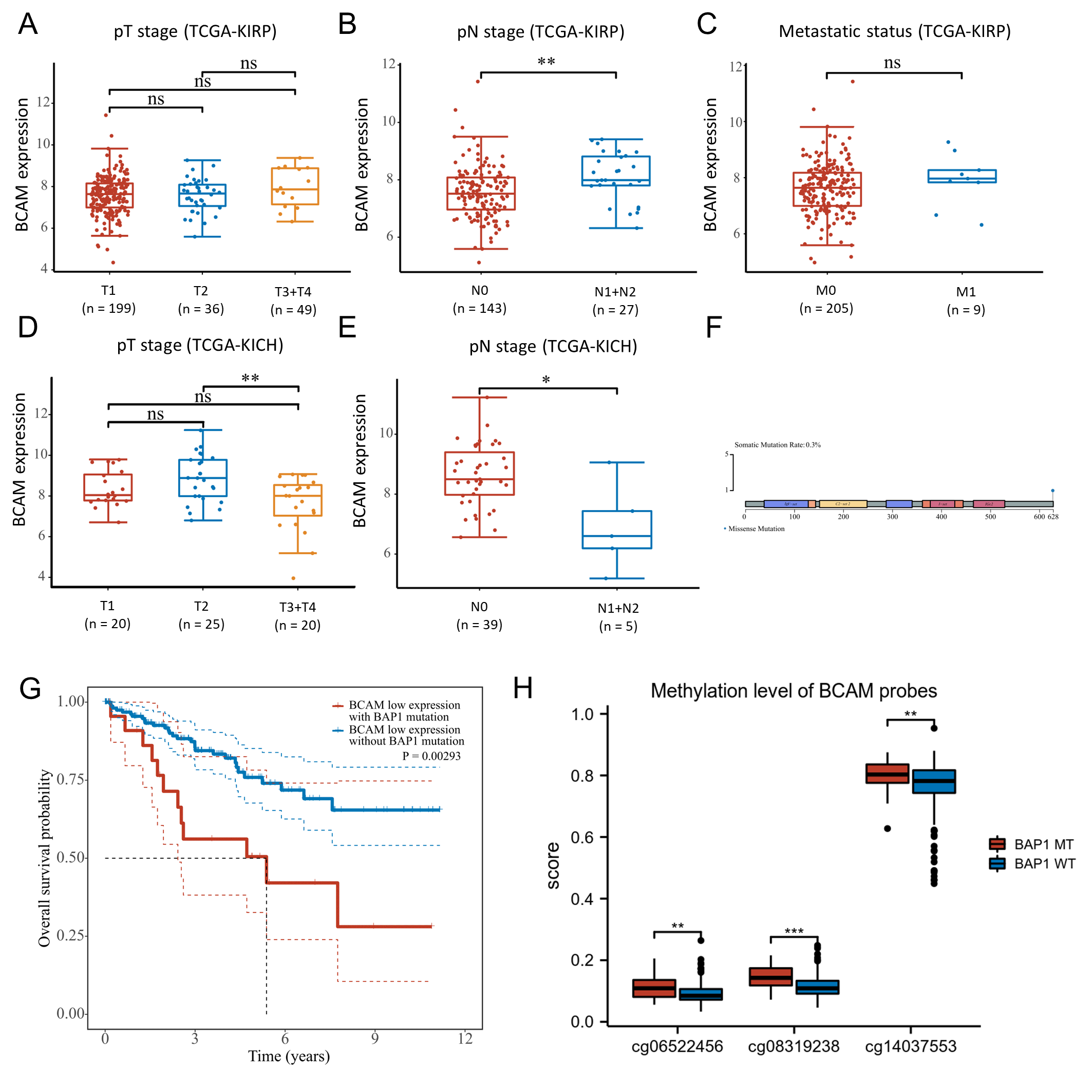

Supplement: Supplementary file 3 — Additional file 3: Fig. S1. A BCAM mRNA expression was not associated with pT stage in pRCC. B BCAM mRNA expression was associated with pN stage in pRCC. C BCAM mRNA expression was not associated with metastatic status in pRCC. D BCAM mRNA expression was not associated with pT stage in chRCC. E BCAM mRNA expression was associated with pN stage in chRCC. *p < 0.05, **p < 0.01. F Mutation distribution and protein domains for BCAM gene in ccRCC with the labeled recurrent hotspots. G Kaplan-Meier analysis of the association between BAP1 mutation status and OS in the BCAM-low subgroup. H The methylation level of BCAM probes between ccRCC with different BAP1 mutation status. MT = mutation type, WT = wild type. **p < 0.01, ***p < 0.001. [file 13148_2022_1319_MOESM3_ESM.tif]

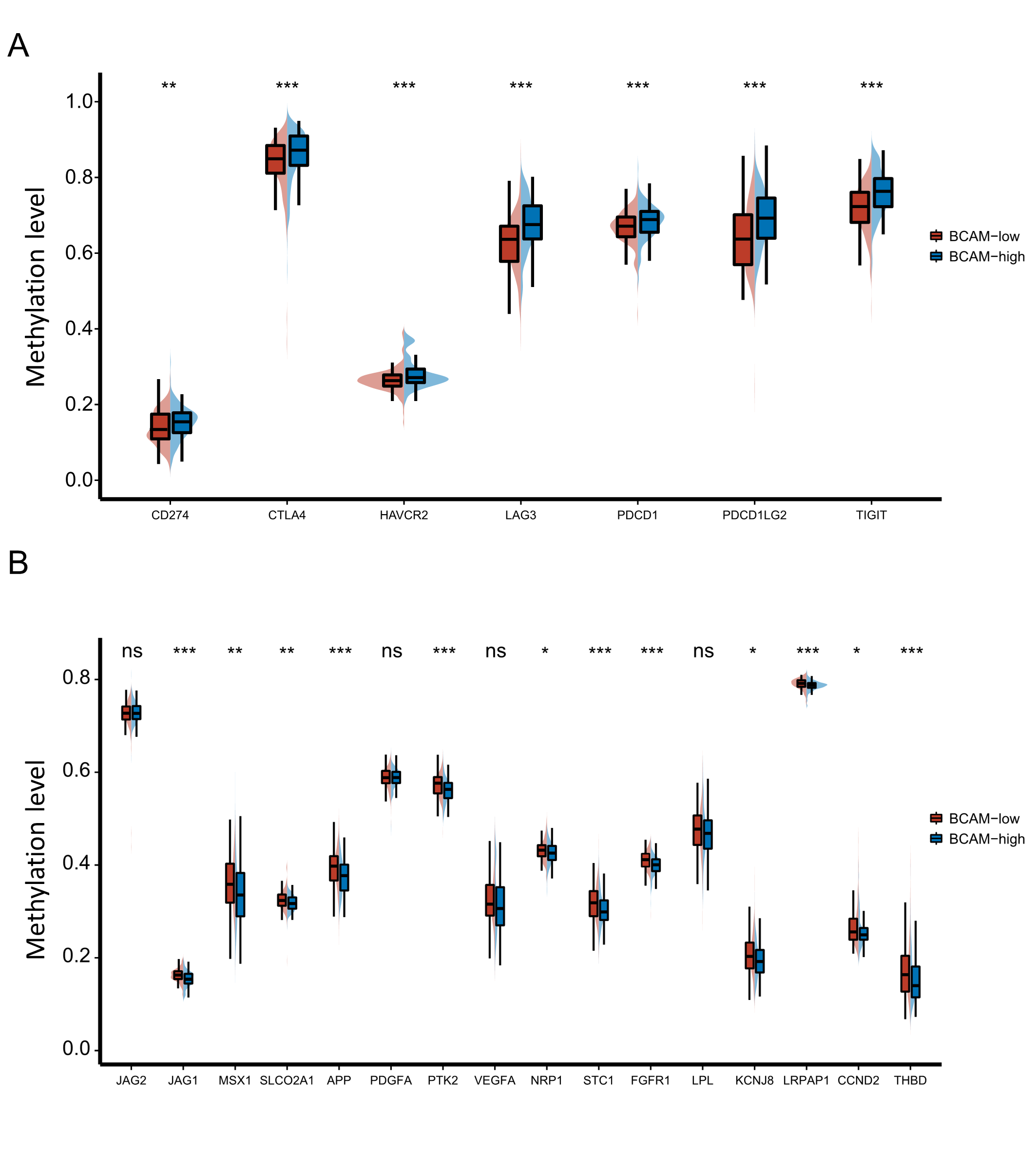

Supplement: Supplementary file 5 — Additional file 5: Fig. S2. A Methylation level of immune checkpoints with higher expression in the BCAM-low subgroup between the BCAM-low and BCAM-high subgroups. B Methylation level of angiogenesis-related genes which were significantly upregulated in the BCAM-high subgroup (p < 0.001) between the BCAM-low and BCAM-high subgroups. *p < 0.05, **p < 0.01, ***p < 0.001. [file 13148_2022_1319_MOESM5_ESM.tif]
