# Supplementary material for: Integrated multi-omics analyses reveal that BCAM is associated with epigenetic modification and tumor microenvironment subtypes of clear cell renal cell carcinoma
Source: Clin Epigenetics. 2022 Aug 8;14:99. doi: 10.1186/s13148-022-01319-2 (PMC9361577; doi:10.1186/s13148-022-01319-2)
Supplement: Supplementary file 6 — Additional file 6: Methods of clinicopathology and prognosis analysis, functional and pathway enrichment analysis, immune infiltration and immune checkpoint analysis. [file 13148_2022_1319_MOESM6_ESM.docx]

**Clinicopathology and prognosis analysis**

The association between *BCAM* mRNA expression and various clinical features of ccRCC, pRCC and chRCC were analyzed, including age, gender, pT stage, pN stage, metastatic status, tumor grading. It was considered that there was statistical difference if p < 0.05. Then, the *BCAM* proteomic expression difference in ccRCC was also explored among different subgroups. Ultimately, we conducted the overall survival (OS) analysis to assess the potential association between the expression status of the *BCAM* gene and the prognosis of ccRCC, pRCC, chRCC respectively using GEPIA (Gene Expression Profiling Interactive Analysis).^1^ The results were visualized by plotting the Kaplan-Meier curve, and we identified the median value as the cutoff between high expression and low expression groups.

**Functional and pathway enrichment analysis**

In order to better comprehend the biological functions and signaling pathways correlated with BCAM gene expression, first, the “Limma” package was used to analyze the differential expression of mRNAs between the *BCAM*-low and *BCAM*-high subgroups. Volcano plot was constructed to reveal all the over-expressed and down-expressed mRNAs with statistical significance. Adjusted p < 0.05 and fold change = 1.5 were defined as the thresholds. Due to the large number of differential genes, we also plotted a heatmap to shown the 50 up-regulated genes and 50 down-regulated genes with the largest differential changes, in which different colors represent the expression trend in ccRCC samples. After that, the transcriptome data of TCGA ccRCC were analyzed by performing Gene Ontology (GO) enrichment analysis and Kyoto Encyclopedia of Gene and Genomes (KEGG) pathway enrichment analysis. For GO enrichment analysis, biological process, cellular component and molecular function were all taken into account. We employed the “ClusterProfiler” package to analyze the GO function of differential genes between the *BCAM*-low and *BCAM*-high subgroups and enrich the KEGG pathway, p < 0.05 or FDR < 0.05 was considered to be an enriched pathway.

**Immune infiltration and immune checkpoint analysis**

To further explore the potential correlation between *BCAM* expression and immunological conditions in TME in ccRCC, we then used the “immunedeconv” package to observe the difference of immune cell score distribution between the *BCAM*-low and *BCAM*-high subgroups. xCELL algorithm was selected for analyzing. A heatmap was plotted and Wilcox test was exploited to determine the significance of the two groups. In addition, the proportions of various immune cells in microenvironment of ccRCC were calculated and exhibited. Furthermore, according to different immune characteristics, ccRCC samples in CPTAC database were divided into metabolic immune-desert subgroup, VEGF immune desert subgroup, CD8- inflamed subgroup and CD8+ inflamed subgroup, and the potential association between the 4 immune subgroups and the RNA and protein expression and methylation level of *BCAM* was analyzed.^2^ Ultimately, the expression values of immune checkpoint SIGLEC15, TIGIT, CD274, HAVCR2, PDCD1, CTLA4, LAG3 and PDCD1LG2 genes in the *BCAM*-low and *BCAM*-high subgroups were extracted using “ggplot2” package.

1. Tang, Z. *et al.* GEPIA: a web server for cancer and normal gene expression profiling and interactive analyses. *Nucleic Acids Research* **45**, W98–W102 (2017).

2. Clark, D. J. *et al.* Integrated Proteogenomic Characterization of Clear Cell Renal Cell Carcinoma. *Cell* **179**, 964-983.e31 (2019).
